# Surface Thermodynamic Properties of Poly Lactic Acid by Inverse Gas Chromatography

**DOI:** 10.3390/biomimetics9050268

**Published:** 2024-04-28

**Authors:** Tayssir Hamieh

**Affiliations:** 1 Faculty of Science and Engineering, Maastricht University, P.O. Box 616, 6200 MD Maastricht, The Netherlands; t.hamieh@maastrichtuniversity.nl; Tel.: +31-6-5723-9324; 2 Laboratory of Materials, Catalysis, Environment and Analytical Methods (MCEMA), Faculty of Sciences, Lebanese University, Hadath P.O. Box 6573, Lebanon

**Keywords:** 3D/4D printing, adhesion, London and polar surface energies, glass transition, enthalpic and entropic Lewis’s acid–base constants, acid and base surface energies

## Abstract

Poly lactic acid (PLA) is one of the most commonly used bio-derived thermoplastic polymers in 3D and 4D printing applications. The determination of PLA surface properties is of capital importance in 3D/4D printing technology. The surface thermodynamic properties of PLA polymers were determined using the inverse gas chromatography (IGC) technique at infinite dilution. The determination of the retention volume of polar and non-polar molecules adsorbed on the PLA particles filling the column allowed us to obtain the dispersive, polar, and Lewis’s acid–base surface properties at different temperatures from 40 °C to 100 °C. The applied surface method was based on our recent model that used the London dispersion equation, the new chromatographic parameter function of the deformation polarizability, and the harmonic mean of the ionization energies of the PLA polymer and organic molecules. The application of this new method led to the determination of the dispersive and polar free surface energy of the adsorption of molecules on the polymeric material, as well as the glass transition and the Lewis acid–base constants. Four interval temperatures were distinguished, showing four zones of variations in the surface properties of PLA as a function of the temperature before and after the glass transition. The acid–base parameters of PLA strongly depend on the temperature. The accurate determination of the dispersive and polar surface physicochemical properties of PLA led to the work of adhesion of the polar organic solvents adsorbed on PLA. These results can be very useful for achieving reliable and functional 3D and 4D printed components.

## 1. Introduction

Since the first description of three-dimensional (3D) printing in the 1980s, many publications have been devoted to additive manufacturing, also known as 3D printing, which has been extremely developed in many applications such as engineering, materials science, physics and astronomy, computer science, chemistry, mathematics, genetics, and molecular biology [1]. In 3D printing, the three-dimensional object is created from a digital model by adding material, typically in successive layers, on the contrary to traditional manufacturing technologies, such as machining, grinding, and casting, where molten material is filled in a mold to create a product [1,2,3,4].

The increasing evolution of the spatial technology of 3D printing led to fourth-dimensional printing (4D printing) by considering the fourth dimension of time to modulate one or more properties of the 3D-printed objects with the help of smart materials that can control the application of any external stimulus implying light, water, self-diagnostic, heating, pressure, and shape-changing effects [1,2,3,4,5,6,7,8,9].

One of the most used materials in 3D/4D printing is poly (lactic acid) (PLA) because of its unique properties such as good appearance, higher transparency, less toxicity, and low thermal expansion, which help reduce the internal stresses caused during cooling [10,11,12,13]. PLA, a bio-derived thermoplastic polymer, is 100% biodegradable polymer with high tensile strength and modulus, and it is easily synthesized from lactic acid obtained from corn, sugarcane, and other biomass. It can be recycled up to eight times and is compostable at the end of life [1,14]. 

The highest tensile and flexural strengths of PLA and the use of its composites with bio-derived reinforcements such as flax, hemp, jute, bamboo, and other natural fibers were widely researched for 3D printing to enhance mechanical properties, reduce material and production costs, and improve the sustainability of manufactured products [14,15,16,17,18,19,20,21,22,23,24,25,26].

PLA, a biodegradable aliphatic polyester, is produced from renewable resources and has received much attention in the research of alternative biodegradable polymers [27,28,29]. This PLA polymer is the most popular polymer in the world and may be processed using standard machines, equipment, and technologies for classic polymers [27,30,31]. PLA shows good biocompatibility and physical properties, such as high mechanical strength, thermoplasticity, and fabricability [27].

The biodegradable PLA polymer is the most used material worldwide for 3D printing [1,14,15,27], and it is very sought after in 4D printing technology. PLA is an excellent bio-derived polymer that is now used as a shape memory polymer in 4D printing applications [20,21,22,23,24,25]. The future of 4D printing bio composites involves multi-disciplinary research to combine design strategies, material properties, stimulus properties, and composite mechanics [1].

The competing mechanical properties and the highest tensile strength of PLA compared to other bio-derived thermoplastics led to a wide utilization of PLA for 3D/4D printing. The best mechanical properties of bio-derived thermoplastics are used in 3D printing. The PLA composites efficiently responded to the requirements in 3D/4D printing applications by improving the mechanical properties and functionalities, such as their ability to be used as a shape memory polymer in 4D printing [1]. The tensile strength and modulus of PLA make it a completely biodegradable polymer.

The thermal, mechanical, and biodegradation properties and glass transition temperature of PLA are necessary to be determined for reinforcement processes and uses in many 3D printing applications by correlating the tensile strength and Young’s modulus of bio-derived fiber reinforcement/PLA to the surface and adhesion properties of PLA [1]. The determination of the interfacial energetic properties of PLA is very suitable to determine the adhesion of the fiber/matrix, which plays a crucial role in the selection of fibers and PLA composites in 3D/4D printing. The accurate determination of the surface physicochemical properties is then required to optimize processing parameters to produce continuously reinforced PLA 3D printed composites with maximum interfacial adhesion to avoid failure due to defects [1,8,32,33,34]. 

The surface properties, the Lewis acid–base parameters, and the dispersive and polar energies of PLA polymers, are very important to be determined in many 3D and 4D applications involving mechanical, adhesion, and surface properties. The determination of the polar and dispersive properties of PLA was based on the literature on different classic chromatographic methods that were proven inaccurate in several studies [35,36,37,38,39,40,41]. Indeed, the London dispersive energy was previously calculated by neglecting the effect of the temperature on the surface area of organic solvents, whereas recent studies have shown an important effect of the temperature on the surface area of molecules, and consequently, the surface properties of PLA had to be corrected in light of the new findings [36,37,38,39].

In this paper, we were interested in determining the surface thermodynamic properties and the various variables of interactions between PLA polymers and other organic molecules. The technique used to study the polymer material was the inverse gas chromatography (IGC) technique at infinite dilution (ID). Our new models [35,36,37,38,39,40,41] were applied to quantify the dispersive and polar interaction energies to understand the behavior of PLA polymer and, therefore, predict the various superficial thermodynamic properties of this 3D/4D printing material in interaction with organic molecules. 

The London dispersive interaction [35,36,37,42] between the solvents and the solid materials was determined by applying the London equation and the notion of polarizabilities and ionization energies of the organic molecules and the polymeric material. This new methodology led to the separation between the dispersive and polar surface free energies of PLA and to the accurate determination of the Lewis enthalpic and entropic acid–base constants, the polar acid and base surface energies, and the glass transition of the PLA polymer. Determining the dispersive and polar surface physicochemical properties of PLA will help determine the adhesion behavior that is essential for achieving reliable and functional 3D and 4Dprinted components.

## 2. Methods and Materials 

In this paper, inverse gas chromatography (IGC) at infinite dilution (ID) was used to determine the net retention time of organic solvents adsorbed on the solid materials [43,44,45,46,47,48,49,50,51,52,53,54,55,56,57,58,59,60,61,62,63,64,65,66]. This resulted in the calculation of the net retention volume Vn of the adsorbed probes and, therefore, the values of the free energy of adsorption $∆G_{a}^{0}$ of organic molecules adsorbed on PLA polymers given by the following equation:

(1) $$∆G_{a}^{0}\left(T\right)=-RTlnVn+K(T)$$

where T is the absolute temperature, R the perfect constant gas, and K(T) is a constant depending on the temperature and the interaction between solvents and PLA.

$∆G_{a}^{0}(T)$ is expressed at any temperature by the summation of the London dispersive energy $∆G_{a}^{d}(T)$ and the polar energy $∆G_{a}^{sp}(T)$:

(2) $$∆G_{a}^{0}\left(T\right)=∆G_{a}^{d}(T)+∆G_{a}^{p}(T)$$

Many methods and molecular models were used in the literature [43,44,45,46,47,48,49,50,51,52,53,54,55,56,57,58,59,60,61,62,63,64,65,66,67] to separate the two dispersive and polar contributions of the free energy of adsorption. It was previously shown [35,36,37] that the best method that gave the most accurate separation between $∆G_{a}^{d}(T)$ and $∆G_{a}^{p}(T)$ was based on the London dispersion interaction energy given by Equation (3):

(3) $$∆G_{a}^{d}\left(T\right)=-\frac{\alpha_{0S}\;}{\;H^{6}}\left[\frac{3N}{{2\left(4\pi \varepsilon_{0}\right)}^{2}}\left(\frac{\varepsilon_{S}\;\varepsilon_{X}}{\left(\varepsilon_{S}+\varepsilon_{X}\right)}\alpha_{0X}\right)\right]$$

where N is the Avogadro number, $\varepsilon_{0}$ the dielectric constant of vacuum, $\alpha_{0S}\;$and $\alpha_{0X}$ are the respective deformation polarizabilities of the solid material denoted by S and the organic molecule denoted by X, separated by a distance H, and $\varepsilon_{S}$ and $\varepsilon_{X}$ are their corresponding ionization energies.

By combining Equations (1)–(3), Equation (4) was obtained:

(4) $$RTlnVn=\frac{\alpha_{0S}\;}{\;H^{6}}\left[\frac{3N}{{2\left(4\pi \varepsilon_{0}\right)}^{2}}\left(\frac{\varepsilon_{S}\;\varepsilon_{X}}{\left(\varepsilon_{S}+\varepsilon_{X}\right)}\alpha_{0X}\right)\right]-∆G_{a}^{sp}\left(T\right)+K\left(T\right)$$

The chosen interaction parameter $P_{SX}$ was given by Equation (5):

(5) $$P_{SX}=\frac{\varepsilon_{S}\;\varepsilon_{X}}{\left(\varepsilon_{S}+\varepsilon_{X}\right)}\alpha_{0X}$$

For non-polar molecules such as n-alkanes, the representation of RTlnVn as a function of $\left[\frac{3N}{{2\left(4\pi \varepsilon_{0}\right)}^{2}}\left(\frac{\varepsilon_{S}\;\varepsilon_{X}}{\left(\varepsilon_{S}+\varepsilon_{X}\right)}\alpha_{0X}\right)\right]$ of adsorbed molecules is given by Equation (6):

(6) $$RTlnVn\left(non-polar\right)=A\left[\frac{3N}{{2\left(4\pi \varepsilon_{0}\right)}^{2}}P_{SX}\left(non-polar\right)\right]-K(T)$$

where A is the slope of the non-polar straight line given by the following:

(7) $$A=\frac{\alpha_{0S}\;}{\;H^{6}}$$

For a polar molecule adsorbed on a PLA polymer, the geometric point representing the polar probe will be located outside the straight line of n-alkanes, and the distance between the polar point and this straight line will be equal to $∆G_{a}^{p}\left(polar\right)$ of the polar molecule at a chosen temperature.

(8) $$∆G_{a}^{p}\left(T,\;polar\right)=RTlnVn\;\left(T,\;polar\right)-A\left[\frac{3N}{{2\left(4\pi \varepsilon_{0}\right)}^{2}}P_{SX}\left(polar\right)\right]+K(T)$$

In the case of linear variations of $∆G_{a}^{p}\left(T\right)$ of polar probes as a function of the temperature, it is possible to deduce the specific enthalpy $\left(-∆H_{a}^{p}\right)$ and entropy $\left(-∆S_{a}^{p}\right)$ of polar probes adsorbed on a PLA polymer using the classic thermodynamic relation (9)):

(9) $$∆G_{a}^{p}\left(T\right)=∆H_{a}^{p}-T∆S_{a}^{p}$$

The determination of $\left(-∆H_{a}^{p}\right)$ and $\left(-∆S_{a}^{p}\right)$ of adsorbed polar molecules leads to the characterization of the Lewis’s acid–base properties of the PLA polymer by its enthalpic (*K_A_*, *K_D_)* and entropic ($\omega_{A}$, $\omega_{D}$) acid–base constants using the following relations:

(10) $$\left\{\begin{array}{c} \left(-∆H^{p}\right)=\;K_{A}\times DN^{\prime }+K_{D}\times AN^{\prime }\;\;\\ \left(-∆S_{a}^{p}\right)=\omega_{A}\times {DN}^{\prime }+\omega_{D}\times {AN}^{\prime } \end{array}\right.$$

where $DN^{\prime }$ and $AN^{\prime }$ are, respectively, the corrected electron donor and acceptor numbers of the polar molecule [68,69].

The experimental results showed that the relation (10) were not always satisfied. In similar cases, other relation (11) were proposed in the literature [39,41,70], taking into consideration the amphoteric coupling constants $K_{CC}$ and $\omega_{CC}$ of solid materials:

(11) $$\left\{\begin{array}{c} \left(-∆H^{p}\right)=\;K_{A}\times DN^{\prime }+K_{D}\times AN^{\prime }-K_{CC}\times AN^{\prime }\times DN^{\prime }\\ \left(-∆S_{a}^{p}\right)=\omega_{A}\times {DN}^{\prime }+\omega_{D}\times {AN}^{\prime }-\omega_{CC}\times AN^{\prime }\times DN^{\prime } \end{array}\right.$$

Relation (11) can be written as follows:

(12) $$\left\{\begin{array}{c} a_{i}\;K_{A}+K_{D}-{b_{i}\;K}_{CC}={\left(c_{H}\right)}_{i}\;\\ a_{i}\;\omega_{A}+\omega_{D}-b_{i}\;\omega_{CC}={\left(c_{S}\right)}_{i}\; \end{array}\right.$$

where $a_{i}$, $b_{i}$, ${\left(c_{H}\right)}_{i}$, and ${\left(c_{S}\right)}_{i}$, relative to the adsorbed polar molecule denoted by i, are the known experimental values given by Equation (13), whereas $K_{D}$, $K_{A}$, $K_{CC}$, $\omega_{A}$, $\omega_{D}$, and $\omega_{CC}$ are the unknown quantities of the problem (12).

(13) $$\left\{\begin{array}{c} a_{i}={\left(\frac{DN^{\prime }}{AN^{\prime }}\right)}_{i}\\ b_{i}={\left(DN^{\prime }\right)}_{i}\\ {\left(c_{H}\right)}_{i}={\left(\frac{\left(-∆H^{p}\right)}{AN^{\prime }}\right)\;}_{i}\\ {\left(c_{S}\right)}_{i}={\left(\frac{\left(-∆S^{p}\right)}{AN^{\prime }}\right)}_{i} \end{array}\right.$$

The unique solution of the system (12) can be obtained if the number n of polar solvents satisfies n≥3 using the least squares method. The obtained solution $\left(K_{D};\;K_{A};K_{CC}\right)$ or $\left(\omega_{D};\;\omega_{A};\;\omega_{CC}\right)$ thus minimizes the sum of the squares of the residuals.

### Materials

PLA polymers with a molecular weight of 40,000 and all organic solvents (highly pure grade (i.e., 99%) were purchased from Sigma-Aldrich (Beirut, Lebanon). The various non-polar molecules used in this study were n-alkanes (pentane, hexane, heptane, octane, and nonane); acidic (dichloromethane), amphoteric (acetone and toluene); and basic solvents (ethyl acetate and tetrahydrofuran (THF)). The PLA particles of sizes between 100 and 250 μm were introduced into a stainless steel column, which was 30 cm long and had an internal diameter of 5 mm. A mass of 1 g of PLA was used to fill the chromatographic column. The column filled with the sample was conditioned at 120 °C for 12 h to remove any impurities. Helium was used as carrier gas with a flow rate equal to 25 mL/min. The IGC measurements at infinite dilution were carried out with a DELSI GC 121 FB Chromatograph from Delsi Instruments (Suresnes, France) equipped with a flame ionization detector of high sensitivity. The injector and detector temperatures were maintained at 180 °C during the experiments. To achieve an infinite dilution approach in linear condition gas chromatography, 0.1 µL of each probe was injected with 1 µL Hamilton syringes. The interactions between probe molecules could be neglected, and only the interactions between the surface of the solid and an isolated probe molecule were important. The column temperatures ranged from 40 to 100 °C, and they varied in 5 °C steps. Each probe injection was repeated three times, and the average retention time was used for the calculation of the retention volume. The standard deviation was less than 1% in all of the measurements.

## 3. Results

### 3.1. London Dispersive Component of Surface Energy of PLA

By using the same procedure developed in previous studies [35,36] and varying the temperature of the chromatographic column containing the PLA particles, the IGC technique allowed us to obtain the net retention times of the various solvents adsorbed on the PLA polymer. This led to the net retention volumes of injected probes and, therefore, the values of RTlnVn of adsorbed organic molecules. The experimental results are given in Tables S1 and S2 (Supporting Materials).

**Figure 1:**
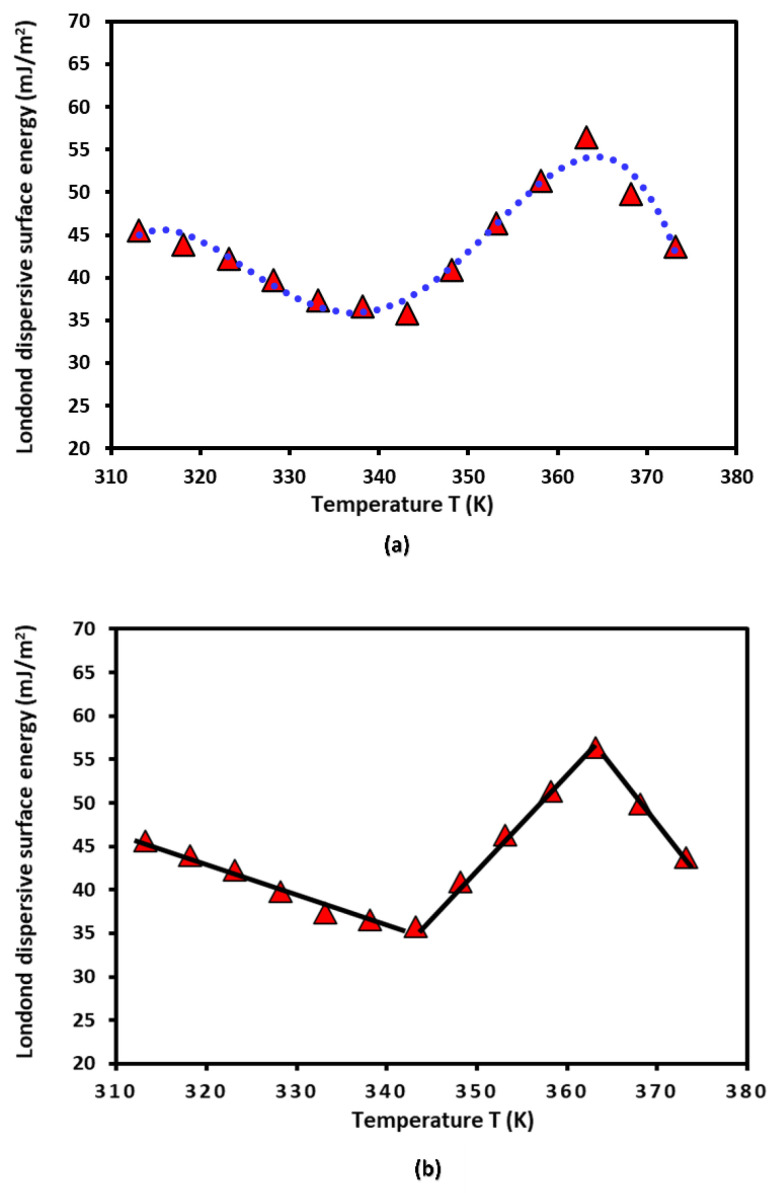
Evolution of $\gamma_{s}^{d}\;(mJ/m^{2})$ of the PLA polymer as a function of the temperature *T* (K) using the Hamieh thermal model. (**a**) Non-linear evolution of $\gamma_{s}^{d}(T)$ and (**b**) three linear variations.

The London dispersive component $\gamma_{s}^{d}(T)$ of the surface energy of PLA polymer was obtained by applying the Hamieh thermal model [37,38,39,70,71]. The variation in $\gamma_{s}^{d}(T)$ of the PLA polymer as a function of the temperature plotted in Figure 1. 

A non-linear evolution of $\gamma_{s}^{d}(T)$ was observed (Figure 1a), showing an important change in the thermodynamic properties of PLA when the temperature varied. However, three linear variations were distinguished (Figure 1b), which were characterized by a decrease in $\gamma_{s}^{d}(T)$ in the temperature interval [313.15 K, 333.15K], given in Equation (14):

(14) $$\gamma_{s}^{d}\left(T\right)=-0.384\;T+165.87$$

Passing by a minimum of the London dispersive surface energy equal to 35.8 mJ/m^2^ at T=343.15 K, followed by an increase variation of $\gamma_{s}^{d}\;(T)$ to reach a maximum equal to 56.4 mJ/m^2^ at T=363.15 K, with the following straight line Equation (15):

(15) $$\gamma_{s}^{d}\left(T\right)=1.030\;T+317.68$$

And finally, a decrease characterized by Equation (16):

(16) $$\gamma_{s}^{d}\left(T\right)=-1.272\;T+518.31$$

This interesting result highlighted the possible glass transition temperature of PLA around $T_{g}=343.15\;K$ (70 °C) explained by the change in the variations of $\gamma_{s}^{d}(T)$ of PLA, before and after $T_{g}$, when the temperature increased.

### 3.2. Polar Surface Free Energy of PLA Polymer

All thermodynamic surface properties of PLA polymers were obtained using our new method of the harmonic mean of the ionization energies and the deformation polarizability of particles. Table 1 and Table 2, respectively, present the values of deformation polarizability, the harmonic mean of the ionization energies, and the parameter $P_{PLA-X}$ of the various organic molecules in interaction with the PLA polymer. The Handbook of Physics and Chemistry [72] was used to determine the parameters of the different solvents.

The obtained experimental results (Tables S1 and S2), the values in Table 1 and Table 2, and the Equations (2), (4), and (8) allowed us to calculate the polar free surface energy ($-∆G_{a}^{sp}\left(T\right)$) of the polar solvents adsorbed on the PLA polymer as a function of the temperature *T* (Table 3).

**Figure 2:**
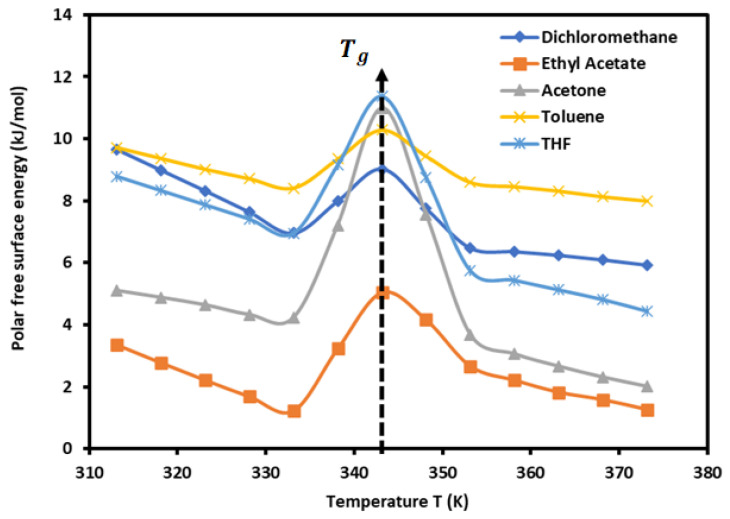
Variations in the polar free surface energy ($-∆G_{a}^{sp}\left(T\right)$) of polar solvents adsorbed on PLA polymers as a function of temperature.

The results in Table 3 show an amphoteric behavior of PLA with a stronger basic character, which is clearly shown by the high values of $-∆G_{a}^{sp}\left(T\right)$ of dichloromethane, the most acidic solvent among the five used polar molecules, then traducing the important interaction energy with PLA. It can be observed in Table 3 that the variations in $-∆G_{a}^{sp}\left(T\right)\;$for all polar molecules are not linear. The curves of $-∆G_{a}^{sp}\left(T\right)$ of polar solvents adsorbed on PLA in Figure 2 prove this non-linearity against the temperature, with a maximum temperature around 343.15 K confirming the presence of the glass temperature of PLA, which was previously shown with the variations in the London dispersive surface energy $\gamma_{s}^{d}\left(T\right)$ as a function of the temperature.

The results plotted in Figure 2 show an important non-monotonous variation in the polar free surface energy as a function of temperature due to the effect of the glass transition on the surface properties of the polymer. It has been proven in a previous work [73] that the presence of a glass transition temperature for a polymer affects the physicochemical properties and the dispersive and polar free energies of the solid surfaces. The change in the values of the free energy and enthalpy of adsorption of polar solvents on PLA will directly affect the acid–base constants of the polymer. In general, the free energy of adsorption decreases as a function of the temperature. However, in the case of a polymer with a glass transition, there is a broken variation of the free energy, enthalpy, entropy, and all parameters of adsorption. This necessarily causes a variation in the acid–base parameters of the polymer. In a previous study [73], the same variations were observed in the case of acrylate cellulose. Adsorption is characterized by a negative value of the enthalpy of adsorption, while a positive value is obtained in the desorption case. 

### 3.3. Lewis’s Acid–Base Constants of PLA

The curves of ($-∆G_{a}^{sp}\left(T\right)$) of polar molecules drawn in Figure 2 show four different temperature intervals in which the variations of ($-∆G_{a}^{sp}\left(T\right)$) are represented by a straight line with an excellent linear regression coefficient equal to 0.9990. The results are given in Table 4 for the different equations.

The values of the polar enthalpy ($-∆H_{a}^{p})$ and entropy ($-∆S_{a}^{p})$ of the polar molecules adsorbed on PLA polymers were deduced from the equations of $∆G_{a}^{sp}\left(T\right)$ in Table 4. The values of these polar thermodynamic parameters are given in Table 5.

Table 5 allowed us to draw in Figure 4 the curves of ($-∆H_{a}^{p}(T)$) and the entropy ($-∆S_{a}^{p}(T)$) of polar molecules as a function of the temperature.

**Figure 3:**
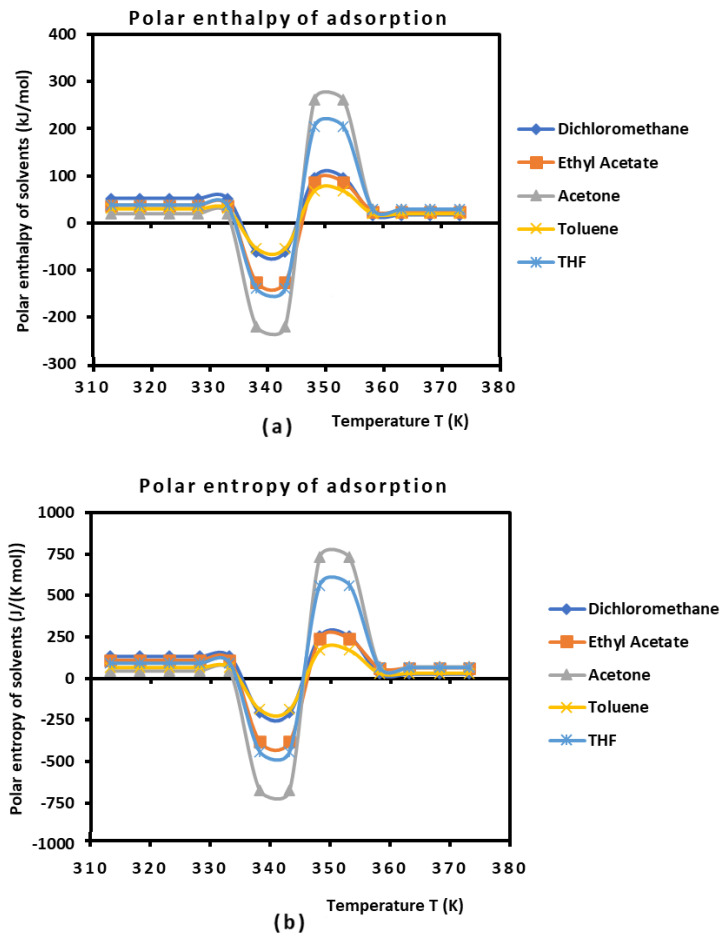
Variations of ($-∆H_{a}^{p}(T)$) (**a**) and entropy ($-∆S_{a}^{p}(T)$) (**b**) of polar molecules adsorbed on PLA polymers as a function of the temperature.

It was observed that the curves in Figure 3 present positive values of polar enthalpy and entropy of polar solvents, before and after the glass transition temperature, highlighting the adsorption phenomenon, whereas desorption was observed during the transition process, showing a repulsive interaction around the glass transition. These types of variations in the acid–base parameters as a function of the temperature have also been observed in other studies [73]. An interesting result can be seen in Figure 3. It concerned the values of ($-∆H_{a}^{p}(T)$) and the entropy ($-∆S_{a}^{p}(T)$) of polar solvents adsorbed on PLAs. Indeed, the variations in these surface parameters are constant for the temperature intervals (T<333.15 K and T>358.15 K) located far from the glass transition ($T_{g}=343.15\;K)$. This will strongly affect the Lewis’s acid–base parameters of PLAs against temperature.

Using the empirical relation (10) and the results in Table 5 and Figure 3, the Lewis enthalpic and entropic acid–base constants, $K_{A}$, $K_{D},\;\omega_{A}$, and $\omega_{D}\;$of PLA polymers, as a function of the temperature with their ratios are given in Table 6.

**Figure 4:**
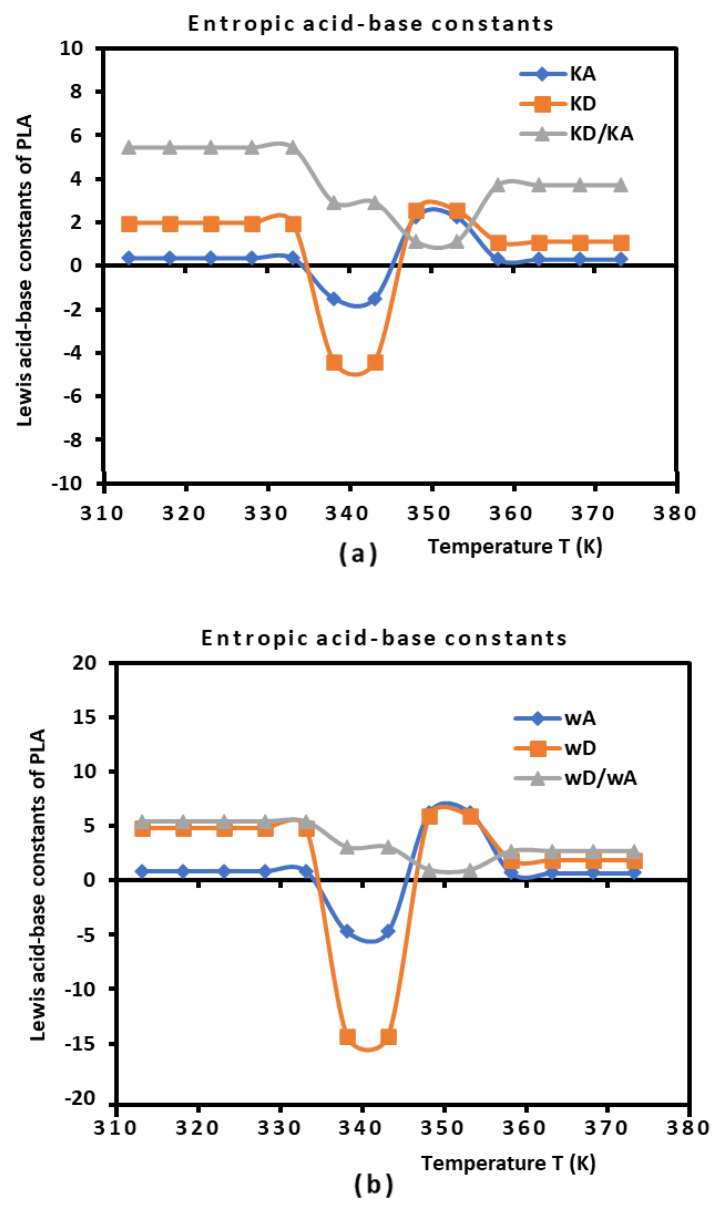
Variations in the Lewis enthalpic acid–base constants $K_{A}$ and $K_{D}$ (**a**) and the Lewis entropic acid base constants $\omega_{A}$ and $\omega_{D}$ (**b**) as a function of the temperature.

**Figure 5:**
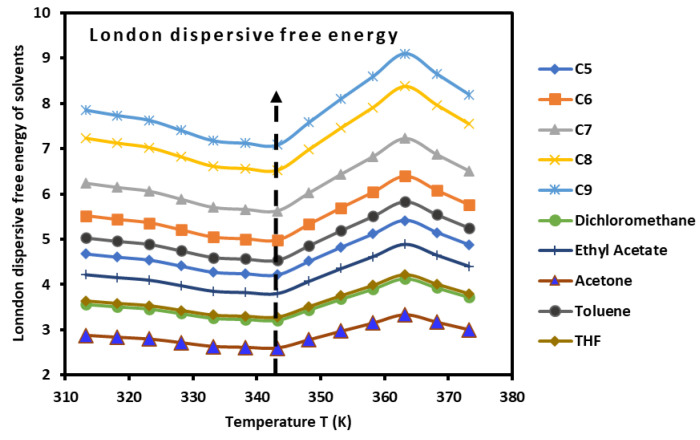
Variations in the London dispersive free energy $∆G_{a}^{d}(T)$ (kJ/mol) of n-alkanes and polar molecules adsorbed on PLA polymers as a function of the temperature.

The results in Table 6 show that the behavior of the PLA surface is 5.5 times more basic than acidic for a temperature less than 333.15 K (Figure 4). The desorption of the polar solvents in the glass transition process led to a neutral surface of the polymer for 333.15 K<T<343.15 K, characterized by negative values of the Lewis acid–base constants $K_{A}$, $K_{D},\;\omega_{A}$, and $\omega_{D}$ of PLA. After the glass transition temperature, two zones were distinguished (Figure 5):

- Stronger amphoteric character of PLAs with the highest values of the Lewis acid–base constants for 343.15 K<T<353.15 K.
- Decreasing amphoteric behavior of the PLA surface with the lowest values of $K_{A}$, $K_{D},\;\omega_{A}$, and $\omega_{D}$ of the polymer for T>353.15 K.

Table 6 and Figure 4 clearly show the stronger basic character of PLA varying with the temperature and decreasing amphoteric behavior for larger temperatures (T>353.15 K). In fact, the observed variations in the Lewis’s acid–base of PLA (Figure 4) showed the same tendence as those of ($-∆H_{a}^{p}(T)$) and entropy ($-∆S_{a}^{p}(T)$) of polar molecules. The variations in all Lewis acid–base variables were constant for T<333.15 K and T>358.15 K, whereas strong non-linear behavior was highlighted around the glass transition in the temperature interval 333.15 K<T<358.15 K.

### 3.4. Correction of the Acid–Base Parameters of PLA

The validity of Equation (10) was not always satisfied, as shown by the values of the linear regression coefficients R^2^ in Table 6. The correction reported by Hamieh et al. in other papers was applied in this work to give more accurate values of the acid–base constants of PLA polymers. Equations (11)–(13) were used alongside procedure developed in a recent paper [35]. The corrected results are presented in Table 7.

The comparison between the results in Table 6 and Table 7 shows that the error committed by neglecting the amphoteric constant reached 25%; however, the tendency of the acid–base behavior of PLA remained the same with the two used methods.

### 3.5. Dispersive and Polar Free Energy of PLA

This new method applied on the PLA polymer using the London dispersion interaction equation resulted in the net separation of the London dispersive free energy $∆G_{a}^{d}(T)$ and the polar free energy $∆G_{a}^{p}(T)$ of interaction between the PLA and the adsorbed organic molecules. By using Equation (3), it was possible to experimentally determine the values of $∆G_{a}^{d}(T)$ of all of the molecules adsorbed on the PLA polymer from the following equation:

(17) $$∆G_{a}^{d}\left(T\right)=A\left[\frac{3N}{{2\left(4\pi \varepsilon_{0}\right)}^{2}}P_{SX}\right]$$

where the values of the parameter A were determined from the experimental results (Table 8).

It was observed that the variations in parameter A given in Table 7 passed through a minimum corresponding exactly to the glass transition temperature $T_{g}=343.15\;K$.

The values of $∆G_{a}^{d}(T)$ of the adsorbed organic molecules were determined (Tables S3 and S4). The obtained results led to drawing the variations in $∆G_{a}^{d}(T)$, which also showed a minimum at the glass transition, as shown in Figure 5. Three linear domains were observed in the curves of the dispersive free energy $∆G_{a}^{d}(T)$ of polar and non-polar adsorbed molecules. These domains were located in the following temperature intervals:

- $T<T_{g}=343.15\;K$, with a positive slope of $∆G_{a}^{d}(T)$.
- $T_{g}<T<363.15\;K$, with a negative slope of $∆G_{a}^{d}(T)$.
- T>363.15 K, with a positive slope of $∆G_{a}^{d}(T)$.

In general, the variations in $∆G_{a}^{d}(T)$ of the adsorbed molecules are linear for many solid materials; however, in the case of the PLA polymer, the presence of the glass transition disrupts this linearity to reach linear variation again.

The experimental determination of the dispersive $∆G_{a}^{d}(T)$ and polar $∆G_{a}^{p}(T)$ energy of interaction allowed us to obtain the total free energy $∆G_{a}^{0}\left(T\right)$ of organic molecules (Table S5 and Figure S1), which also highlighted the presence of the glass transition.

### 3.6. Average Separation Distance H

Table 8 and Equation (7) determine the average separation distance *H* between the PLA surface and the organic molecules as a function of temperature. The results obtained are given in Table 9. These results showed a slight variation in the separation distance when the temperature varies, but it respects the general tendency observed with the other thermodynamic variables, showing a signature at the glass transition temperature reported for H=8.30 Å (Figure S2). The results in Table 9 and Figure S2 show a small increase in the separation distance when the temperature increases until the glass transition T<343.15 K, followed by a slight decrease in H after this temperature. This result confirmed that obtained by the stronger acid–base constants of PLA obtained for T>343.15 K. 

### 3.7. Lewis Acid–Base Surface Energies of PLA

To determine the acid $\gamma_{s}^{+}$ and base $\gamma_{s}^{-}$ surface energy of the PLA polymer, Van Oss’s relation was used [74]:

(18) $$-∆G_{a}^{p}\left(X-Polar\right)=2Na_{X}\left(\sqrt{\gamma_{lX}^{-}\gamma_{s}^{+}}+\sqrt{\gamma_{lX}^{+}\gamma_{s}^{-}}\right)$$

where $\gamma_{lX}^{+}$ and $\gamma_{lX}^{-}$ are the respective acid and base surface energy of the polar molecule X adsorbed on the PLA surface with $a_{X}$, the surface area of the adsorbed solvent.

Using the experimental values relative to ethyl acetate (EA) and dichloromethane (CH_2_Cl_2_), respectively, given by $\gamma_{EA}^{+}=0,\gamma_{EA}^{-}=19.2\;mJ/m^{2}$, and $\gamma_{CH_{2}Cl_{2}}^{+}=5.2\;mJ/m^{2},\gamma_{CH_{2}Cl_{2}}^{-}=0$, it was possible to determine the values of $\gamma_{s}^{+}$ and $\gamma_{s}^{-}$ of the PLA polymer using Equation (19):

(19) $$\left\{\begin{array}{l} \begin{array}{c} \gamma_{s}^{+}=\frac{{\left[∆G_{a}^{sp}\left(T\right)\left(EA\right)\right]}^{2}}{{4N}^{2}{\left[a\left(EA\right)\right]}^{2}\gamma_{EA}^{-}}\; \end{array}\;\;\;\;\;\;\;\;\;\;\;\;\;\;\;\;\\ \gamma_{s}^{-}=\frac{{\left[∆G_{a}^{sp}\left(T\right)\left(CH_{2}Cl_{2}\right)\right]}^{2}}{{4N}^{2}{\left[a\left(CH_{2}Cl_{2}\right)\right]}^{2}\gamma_{CH_{2}Cl_{2}}^{+}}\; \end{array}\right.$$

whereas the acid–base (polar) surface energy $\gamma_{s}^{AB}$ of PLA was obtained from Equation (20):

(20) $$\gamma_{s}^{AB}=2\sqrt{\gamma_{s}^{+}\gamma_{s}^{-}}$$

The results are reported in Table 10 and Figure S3. The variations in the acid and base surface energies versus the temperature showed a decrease in these surface energy parameters and then an increase, reaching a maximum at the glass transition temperature, followed by a final decrease until T=373.15 K. An important basic surface energy $\gamma_{s}^{-}$ of PLA was observed with smaller acidic surface energy.

The results in Table 10 with those relative to the dispersive surface energy of PLAs, previously obtained in this work, led to the determination of the Lifshitz–Van der Waals (LW) surface energy $\gamma_{s}^{LW}$ (Table 11) using Equation (21):

(21) $$\gamma_{s}^{LW}={\gamma_{s}^{d}+\gamma }_{s}^{AB}$$

The determination of the Lifshitz–Van der Waals (LW) surface energy (Table 11) of PLAs showed an important variation as a function of the temperature by presenting a decrease at the glass transition followed by a sudden increase just after the transition, and finishing by a new decrease. The values of $\gamma_{s}^{LW}$ showed the identical variations as those obtained with the free energy, enthalpy, entropy, and acid–base parameters.

### 3.8. Polar Component of the Surface Energy of Polar Molecules

Using the previous results and Equation (22), it is observed that the polar free energy of polar molecules adsorbed on PLA are related to the polar components of the surface energy of the PLA polymer $\gamma_{s}^{p}$ and the polar organic molecules $\gamma_{l}^{p}$.

(22) $$\left\{\begin{array}{c} -∆G_{a}^{p}\left(X\right)=2Na_{X}\;\sqrt{\gamma_{s}^{p}\gamma_{l}^{p}}\\ \;or\;\;\gamma_{l}^{p}=\frac{{\left(-∆G_{a}^{p}\left(X\right)\right)}^{2}}{4N^{2}{a_{X}}^{2}\gamma_{s}^{p}}\;\;\;\;\;\;\;\;\;\;\;\;\; \end{array}\right.$$

$\gamma_{l}^{p}$ of polar molecules was directly obtained from Equation (22). The results are given in Table 12.

Table 12 shows that, among all of the obtained values of the polar components of the surface energy of polar molecules, the stronger $\gamma_{l}^{p}$ values were obtained with dichloromethane, the highest acidic solvent used in this study, once again proving the highest Lewis basicity of PLA polymers. It is shown (Table 12) that the polar component of the surface energy of the various polar organic molecules adsorbed on PLA varied as a function of the temperature. These variations also followed the same trends obtained by the presence of the glass transition, which affected the variations in all of the surface thermodynamic parameters.

### 3.9. Consequences of These Results of the Work of Adhesion

The polar component of the work of adhesion $W_{a}^{p}\left(PLA-X\right)$ of the polar organic molecule X adsorbed on PLA is given by relation 23:

(23) $$W_{a}^{p}\left(PLA-X\right)=2\sqrt{\gamma_{s}^{p}(PLA)\gamma_{l}^{p}(X)}$$

The results in Table 11 and Table 12 led to the determination of $W_{a}^{p}\left(PLA-X\right)$ of the different polar molecules. Table 13 depicts the values of the polar work of adhesion at various temperatures.

The results in Table 13 show that the variations in the work of adhesion of all polar solvents were not linear and that they all admitted a maximum at the glass transition temperature ${(T}_{g}=343.15\;K)$ of PLA. The highest polar work of adhesion was obtained with dichloromethane, respectively, followed by THF, toluene, acetone, and ethyl acetate, again proving the highest Lewis basicity of PLA compared to its Lewis acidity.

## 4. Conclusions

Inverse gas chromatography (IGC) at infinite dilution was used to determine the surface thermodynamic properties of the biodegradable poly lactic acid, which is considered the most interesting material that can be used in 3D printing applications. The new method used was based on the London dispersion interaction equation. This equation took into account the polarizability and the harmonic mean of the ionization energies of PLA polymers and adsorbed organic solvents. The London dispersive energy of PLA materials was determined using the Hamieh thermal model. The free dispersive and polar energies of adsorbed solvents were obtained using the new parameter $P_{SX}$ and the net retention volumes of adsorbed probes from chromatographic measurements. The variations in all thermodynamic parameters of the interaction of organic molecules adsorbed on PLAs highlighted four temperature intervals with linear equations in each interval of temperature. A glass transition temperature of PLA was located at $T_{g}=343.15\;K$. The presence of this transition phenomenon had an important effect on the non-linearity in the domain of the temperature containing the glass transition temperature. This is due to the strong variation of the enthalpic and entropic acid base constants of PLA as a function of temperature. A stronger basic character of the PLA surface was highlighted before and after the glass transition, and a slight variation in the average separation distance between the PLA polymer and the solvents was observed. 

The determination of the various components $\gamma_{s}^{+}$, $\gamma_{s}^{-}$, and $\gamma_{s}^{AB}$ of acid–base surface energies of PLA allowed us to calculate the Lifshitz–Van der Waals surface energy $\gamma_{s}^{LW}$. A dominant basic surface character was shown with the highest value of $\gamma_{s}^{-}$ of PLA. All these surface parameters confirmed the presence of $T_{g}=343.15\;K$ for poly lactic acid.

The application of this new method enabled a net separation between the polar and dispersive free energies and also the determination of the polar components of the surface energy of polar solvents adsorbed on the PLA polymer. These new findings will allow us to make an accurate determination of the polar works of adhesion between the PLA surface and organic molecules. The new values from this work on the adhesion of the solvents and PLA are very important in 3D and 4D printing applications, particularly when the temperature increases. Other studies are now prepared to validate the different results obtained in this work by applying the same methodology to other polymers and, especially, in the case of polymers adsorbed on oxides by varying the tacticity of polymers.

## Figures and Tables

**Table 1:** Values of deformation polarizability $\alpha_{0}$ (respectively, in 10^−30^ m^3^ and in 10^−40^ C m^2^/V) and ionization energy ε (in eV) of the various organic molecules and PLA polymers.

Molecule	$\varepsilon_{X}$$\;\mathrm{or}\;\varepsilon_{S}$ (eV)	$\alpha_{0X}$$\;\mathrm{or}\;\alpha_{0S}$ (in 10^−30^ m^3^)	$\alpha_{0X}$$\;\mathrm{or}\;\alpha_{0S}$ (in 10^−40^ C m^2^/V)
n-pentane	10.28	9.99	11.12
n-hexane	10.13	11.90	13.24
n-heptane	9.93	13.61	15.14
n-octane	9.80	15.90	17.69
n-nonane	9.71	17.36	19.32
n-decane	9.65	19.10	21.25
CH_2_Cl_2_	11.32	7.21	8.02
Tetrahydrofuran	9.38	8.22	9.15
Ethyl acetate	10.01	9.16	10.19
Acetone	9.70	6.37	7.09
Toluene	8.83	11.80	13.13
PLA	14.85	3.35	3.73

**Table 2:** Values of the harmonic mean of the ionization energies of PLA particles and organic solvents (in 10^−19^ J) and the parameter $\frac{3N}{{2\left(4\pi \varepsilon_{0}\right)}^{2}}P_{PLA-X}$ (in 10^−15^ SI unit) for the various organic molecules.

Molecule X	$\frac{\varepsilon_{PLA}\varepsilon_{X}}{\left(\varepsilon_{PLA}+\varepsilon_{X}\right)}$ (in 10^−19^ J)	$\frac{3N}{{2\left(4\pi \varepsilon_{0}\right)}^{2}}P_{PLA-X}$ (in 10^−15^ SI)
C5	6.075	78.831
C6	6.022	93.088
C7	5.951	105.205
C8	5.904	121.937
C9	5.871	132.395
CH_2_Cl_2_	6.423	60.160
Ethyl acetate	5.979	71.147
Acetone	5.869	48.559
Toluene	5.536	84.863
THF	5.749	61.383

**Table 3:** Values of $-∆G_{a}^{p}\left(T\right)$ (in kJ/mol) of polar molecules adsorbed on PLA.

*T* (K)	CH_2_Cl_2_	Ethyl Acetate	Acetone	Toluene	THF
313.15	9.641	3.355	5.097	9.721	8.784
318.15	8.979	2.775	4.875	9.369	8.326
323.15	8.317	2.214	4.634	9.017	7.867
328.15	7.636	1.683	4.321	8.712	7.409
333.15	6.955	1.221	4.230	8.406	6.950
338.15	7.987	3.227	7.213	9.345	9.145
343.15	9.018	5.044	10.980	10.284	11.356
348.15	7.748	4.165	7.543	9.437	8.756
353.15	6.478	2.667	3.675	8.591	5.742
358.15	6.364	2.211	3.054	8.449	5.433
363.15	6.250	1.825	2.654	8.307	5.124
368.15	6.104	1.583	2.305	8.122	4.811
373.15	5.934	1.265	2.005	7.987	4.435

**Table 4:** Equations of $-∆G_{a}^{sp}(T)$ of the polar solvents adsorbed on PLAs for the different temperature intervals.

Solvent	[313.15 K, 333.15 K]	[333.15 K, 343.15 K]	[343.15 K, 353.15 K]	[353.15 K, 373.15 K]
CH_2_Cl_2_	$-∆G_{a}^{sp}=-0.134T+51.718$	$-∆G_{a}^{sp}=0.206T-61.799$	$-∆G_{a}^{sp}=-0.254T+96.18$	$-∆G_{a}^{sp}=-0.029T+16.66$
Ethyl Acetate	$-∆G_{a}^{sp}=-0.107T+36.894$	$-∆G_{a}^{sp}=0.383T-126.11$	$-∆G_{a}^{sp}=-0.238T+86.728$	$-∆G_{a}^{sp}=-0.062T+24.245$
Acetone	$-∆G_{a}^{sp}=-0.046T+19.418$	$-∆G_{a}^{sp}=0.675T-220.79$	$-∆G_{a}^{sp}=-0.731T+261.73$	$-∆G_{a}^{sp}=-0.070T+28.074$
Toluene	$-∆G_{a}^{sp}=-0.066T+30.294$	$-∆G_{a}^{sp}=0.188T-54.185$	$-∆G_{a}^{sp}=-0.169T+68.411$	$-∆G_{a}^{sp}=-0.031T+19.708$
THF	$-∆G_{a}^{sp}=-0.092T+37.500$	$-∆G_{a}^{sp}=0.441T-139.83$	$-∆G_{a}^{sp}=-0.561T+204.07$	$-∆G_{a}^{sp}=-0.065T+28.611$

**Table 5:** Values of polar enthalpy ($-∆H_{a}^{p}\;in\;kJ\;{mol}^{-1}$) and entropy ($-∆S_{a}^{p}\;in\;JK^{-1}\;{mol}^{-1}$) of polar probes adsorbed on PLAs.

Polar enthalpy ($-∆H_{a}^{p}\;in\;kJ\;{mol}^{-1}$)				
Solvent	[313.15 K, 333.15 K]	[333.15 K, 343.15 K]	[343.15 K, 353.15 K]	[353.15 K, 373.15 K]
CH_2_Cl_2_	51.718	−61.799	96.177	16.661
Ethyl acetate	36.894	−126.11	86.728	24.245
Acetone	19.418	−220.79	261.73	28.074
Toluene	30.294	−54.185	68.411	19.708
THF	37.5	−139.83	204.07	28.611
Polar entropy ($-∆S_{a}^{p}\;in\;JK^{-1}{mol}^{-1}$)				
Solvent	[313.15 K, 333.15 K]	[333.15 K, 343.15 K]	[343.15 K, 353.15 K]	[353.15 K, 373.15 K]
CH_2_Cl_2_	134.3	−206.4	254	28.7
Ethyl Acetate	107.2	−382.3	237.7	61.6
Acetone	45.8	−675	730.5	69.9
Toluene	65.8	−187.9	169.4	31.4
THF	91.7	−440.6	561.4	64.7

**Table 6:** Values of the enthalpic acid–base constants $K_{A}$ and $K_{D}\;$(unitless), the entropic acid base constants $\omega_{A}$ and $\omega_{D}$ (unitless), the acid–base ratios of PLA, and the linear regression coefficients R².

Temperature *T* (K)	$K_{A}$	$K_{D}$	$K_{D}$ $/K_{A}$	R²	$10^{3}\;\times \;\omega_{A}$	$10^{3}\;\times \;\omega_{D}$	$\omega_{D}$ $/\omega_{A}$	R²
313.15	0.359	1.963	5.47	0.8168	0.89	4.83	5.43	0.8557
318.15	0.359	1.963	5.47	0.8168	0.89	4.83	5.43	0.8557
323.15	0.359	1.963	5.47	0.8168	0.89	4.83	5.43	0.8557
328.15	0.359	1.963	5.47	0.8168	0.89	4.83	5.43	0.8557
333.15	0.359	1.963	5.47	0.8168	0.89	4.83	5.43	0.8557
338.15	−1.503	−4.417	2.94	0.9779	−4.70	−14.34	3.05	0.9754
343.15	−1.503	−4.417	2.94	0.9779	−4.70	−14.34	3.05	0.9754
348.15	2.259	2.534	1.12	0.9335	6.26	5.92	0.95	0.936
353.15	2.259	2.534	1.12	0.9335	6.26	5.92	0.95	0.936
358.15	0.294	1.096	3.73	0.9099	0.70	1.88	2.68	0.9804
363.15	0.294	1.096	3.73	0.9099	0.70	1.88	2.68	0.9804
368.15	0.294	1.096	3.73	0.9099	0.70	1.88	2.68	0.9804
373.15	0.294	1.096	3.73	0.9099	0.70	1.88	2.68	0.9804

**Table 7:** Values of the corrected acid–base constants $K_{A}$, $K_{D},\;K_{CC},\;\omega_{A}$, $\omega_{D}$, $\omega_{CC}$, and the acid–base ratios of PLA.

Temperature *T* (K)	$K_{A}$	$K_{D}$	$10^{2}\;\times \;K_{CC}$	$K_{D}$ $/K_{A}$	$10^{3}\;\times \;\omega_{A}$	$10^{3}\;\times \;\omega_{D}$	$10^{5}\;\times \;\omega_{CC}$	$\omega_{D}$ $/\omega_{A}$
313.15	0.332	1.651	1.8	4.98	0.83	4.20	3.7	5.03
318.15	0.332	1.651	1.8	4.98	0.83	4.20	3.7	5.03
323.15	0.332	1.651	1.8	4.98	0.83	4.20	3.7	5.03
328.15	0.332	1.651	1.8	4.98	0.83	4.20	3.7	5.03
333.15	0.332	1.651	1.8	4.98	0.83	4.20	3.7	5.03
338.15	−1.503	−4.417	0	2.94	−4.70	−14.34	0	3.05
343.15	−1.503	−4.417	0	2.94	−4.70	−14.34	0	3.05
348.15	2.215	2.040	2.9	0.92	6.16	4.77	0.7	0.77
353.15	2.215	2.040	2.9	0.92	6.16	4.77	0.7	0.77
358.15	0.283	0.972	0.7	3.43	0.69	1.78	0.6	2.58
363.15	0.283	0.972	0.7	3.43	0.69	1.78	0.6	2.58
368.15	0.283	0.972	0.7	3.43	0.69	1.78	0.6	2.58
373.15	0.283	0.972	0.7	3.43	0.69	1.78	0.6	2.58

**Table 8:** Values of the parameter A as a function of the temperature.

Temperature *T* (K)	Parameter A (SI Unit)
313.15	5.93 × 10^−2^
318.15	5.84 × 10^−2^
323.15	5.76 × 10^−2^
328.15	5.59 × 10^−2^
333.15	5.42 × 10^−2^
338.15	5.38 × 10^−2^
343.15	5.35 × 10^−2^
348.15	5.73 × 10^−2^
353.15	6.12 × 10^−2^
358.15	6.49 × 10^−2^
363.15	6.87 × 10^−2^
368.15	6.53 × 10^−2^
373.15	6.19 × 10^−2^

**Table 9:** Values of the average separation distance *H* (in Å) as a function of the temperature.

Temperature *T* (K)	Separation Distance *H* (in Å)
313.15	8.16
318.15	8.18
323.15	8.20
328.15	8.24
333.15	8.29
338.15	8.30
343.15	8.30
348.15	8.21
353.15	8.12
358.15	8.04
363.15	7.97
368.15	8.03
373.15	8.10

**Table 10:** Values of the polar acid and base surface energies $\gamma_{s}^{+}$, $\gamma_{s}^{-}$, and $\gamma_{s}^{AB}$ (mJ/m^2^) of PLAs as a function of the temperature.

*T* (K)	$\gamma_{s}^{-}$	$\gamma_{s}^{+}$	$\gamma_{s}^{AB}$
313.15	50.59	4.38	29.78
318.15	43.66	2.98	22.83
323.15	37.27	1.89	16.78
328.15	31.26	1.09	11.65
333.15	25.80	0.57	7.66
338.15	33.86	3.95	23.14
343.15	42.96	9.61	40.64
348.15	31.55	6.52	28.69
353.15	21.95	2.66	15.28
358.15	21.08	1.82	12.39
363.15	20.23	1.23	9.99
368.15	19.20	0.92	8.42
373.15	18.06	0.59	6.51

**Table 11:** Values of the dispersive energies $\gamma_{s}^{d}$ and the Lifshitz–Van der Waals (LW) surface energy $\gamma_{s}^{LW}$ (mJ/m^2^) of PLAs as a function of the temperature.

*T* (K)	$r_{s}^{d}$	$r_{s}^{LW}$
313.15	45.59	75.37
318.15	43.93	66.75
323.15	42.28	59.06
328.15	39.78	51.43
333.15	37.36	45.02
338.15	36.60	59.74
343.15	35.83	76.47
348.15	40.97	69.66
353.15	46.33	61.61
358.15	51.33	63.72
363.15	56.41	66.40
368.15	49.86	58.28
373.15	43.69	50.20

**Table 12:** Values of $\gamma_{l}^{p}$ (mJ/m^2^) of polar molecules adsorbed on PLAs.

*T* (K)	CH_2_Cl_2_	Ethyl Acetate	Acetone	Toluene	THF
313.15	16.20	1.08	4.53	7.02	8.52
318.15	17.98	0.96	5.38	8.44	9.91
323.15	20.58	0.82	6.58	10.56	11.94
328.15	24.50	0.68	8.19	14.09	15.13
333.15	30.33	0.54	11.87	19.81	20.09
338.15	13.00	1.23	11.37	8.05	11.43
343.15	9.26	1.69	14.92	5.51	9.95
348.15	9.51	1.61	9.92	6.53	8.32
353.15	12.25	1.23	4.40	10.08	6.66
358.15	14.32	1.03	3.72	11.95	7.30
363.15	16.82	0.86	3.47	14.22	7.99
368.15	18.70	0.76	3.08	16.01	8.29
373.15	22.46	0.62	3.00	19.88	9.05

**Table 13:** Values of $W_{a}^{p}\left(PLA-X\right)$ (mJ/m^2^) of polar molecules adsorbed on PLAs.

*T* (K)	CH_2_Cl_2_	Ethyl Acetate	Acetone	Toluene	THF
313.15	43.93	11.36	23.24	28.92	31.86
318.15	40.52	9.35	22.17	27.77	30.08
323.15	37.17	7.42	21.01	26.63	28.31
328.15	33.80	5.61	19.54	25.63	26.56
333.15	30.49	4.05	19.07	24.64	24.81
338.15	34.69	10.65	32.44	27.30	32.52
343.15	38.80	16.56	49.24	29.93	40.23
348.15	33.03	13.61	33.74	27.37	30.90
353.15	27.37	8.67	16.39	24.82	20.18
358.15	26.64	7.15	13.58	24.33	19.02
363.15	25.93	5.87	11.77	23.84	17.87
368.15	25.10	5.07	10.19	23.22	16.72
373.15	24.19	4.03	8.85	22.76	15.35

## Data Availability

The data presented in this study are available in article and supplementary materials.

## Supplementary Materials

The following supporting information can be downloaded at https://www.mdpi.com/article/10.3390/biomimetics9050268/s1. Table S1. Values of −RTlnVn (in kJ/mol) of the various non-polar solvents adsorbed on PLA polymer as a function of temperature. Table S2. Values of −RTlnVn (in kJ/mol) of the various polar solvents adsorbed on PLA polymer as a function of the temperature. Table S3. Values of −$∆G_{a}^{d}(T)$ (kJ/mol) of the various non-polar solvents adsorbed on PLA polymer as a function of temperature. Table S4. Values of −$∆G_{a}^{d}(T)$ (kJ/mol) of the various polar solvents adsorbed on PLA polymer as a function of temperature. Table S5. Values of −$∆G_{a}^{0}(T)$ (kJ/mol) of the various polar solvents adsorbed on PLA polymer as a function of temperature. Figure S1. Variations in the total free energy $∆G_{a}^{d}(T)$ (kJ/mol) of polar molecules adsorbed on PLA polymer as a function of temperature. Figure S2. Variations in the average separation distance *H* (in Å) as a function of temperature. Figure S3. Variations in the polar acid and base surface energies $\gamma_{s}^{+}$, $\gamma_{s}^{-}$, and $\gamma_{s}^{AB}$ (mJ/m^2^) of PLA as a function of temperature.
