# Supplementary material for: Surface Thermodynamic Properties of Poly Lactic Acid by Inverse Gas Chromatography
Source: Biomimetics (Basel). 2024 Apr 28;9(5):268. doi: 10.3390/biomimetics9050268 (PMC11117825; doi:10.3390/biomimetics9050268)
Supplement: Supplementary file 1 [file biomimetics-09-00268-s001.zip › biomimetics-2980218-supplementary.pdf]

Supplementary Materials

Surface thermodynamic properties of poly lactic acid by inverse gas chromatography

Tayssir Hamieh[1,2]

[1]Faculty of Science and Engineering, Maastricht University, P.O. Box 616, 6200 MD Maastricht, Netherlands, E-mail: t.hamieh@maastrichtuniversity.nl
[2]Laboratory of Materials, Catalysis, Environment and Analytical Methods Laboratory (MCEMA), Faculty of Sciences, Lebanese University, 1533, Hadath, Lebanon

Table S1. Values of $-RTlnVn$ (in kJ/mol) of the various non-polar solvents adsorbed on PLA polymer as a function of the temperature.

T(K)	C5	C6	C7	C8	C9
313.15	4.521	3.618	2.498	1.780	1.407
318.15	4.618	3.702	2.630	1.902	1.545
323.15	4.714	3.785	2.763	2.025	1.683
328.15	4.723	3.912	2.888	2.156	1.804
333.15	4.732	4.040	3.014	2.288	1.926
338.15	4.780	4.127	3.074	2.329	2.022
343.15	4.828	4.213	3.135	2.369	2.118
348.15	5.010	4.410	3.287	2.411	2.116
353.15	5.193	4.606	3.439	2.454	2.113
358.15	5.454	4.792	3.630	2.530	2.179
363.15	5.714	4.979	3.822	2.607	2.244
368.15	5.684	4.901	4.034	2.718	2.356
373.15	5.654	4.823	4.247	2.829	2.468

Table S2. Values of $-RTlnVn$ (in kJ/mol) of the various polar solvents adsorbed on PLA polymer as a function of the temperature.

T(K)	CH_2Cl_2	Ethyl Acetate	Acetone	Toluene	THF
313.15	-4.141	1.494	1.092	-5.685	-3.356
318.15	-3.402	2.161	1.380	-5.234	-2.820
323.15	-2.669	2.801	1.682	-4.792	-2.290
328.15	-1.964	3.374	1.999	-4.421	-1.806
333.15	-1.260	3.879	2.094	-4.049	-1.321

338.15	-2.236	1.932	-0.839	-4.924	-3.461
343.15	-3.219	0.167	-4.560	-5.807	-5.622
348.15	-1.653	1.301	-0.783	-4.758	-2.731
353.15	-0.093	3.046	3.420	-3.717	0.568
358.15	0.352	3.792	4.415	-3.337	1.203
363.15	0.789	4.459	5.182	-2.966	1.831
368.15	0.846	4.649	5.403	-2.786	2.058
373.15	0.926	4.915	5.573	-2.656	2.349

Table S3. Values of -$\Delta G_a^d(T)$ (kJ/mol) of the various non-polar solvents adsorbed on PLA polymer as a function of the temperature.

T(K)	C5	C6	C7	C8	C9
313.15	4.675	5.520	6.239	7.231	7.851
318.15	4.604	5.436	6.144	7.121	7.732
323.15	4.541	5.362	6.060	7.024	7.626
328.15	4.407	5.204	5.881	6.816	7.401
333.15	4.273	5.045	5.702	6.609	7.176
338.15	4.241	5.008	5.660	6.560	7.123
343.15	4.217	4.980	5.628	6.524	7.083
348.15	4.517	5.334	6.028	6.987	7.586
353.15	4.824	5.697	6.439	7.463	8.103
358.15	5.116	6.041	6.828	7.914	8.592
363.15	5.416	6.395	7.228	8.377	9.096
368.15	5.148	6.079	6.870	7.962	8.645
373.15	4.880	5.762	6.512	7.548	8.195

Table S4. Values of -$\Delta G_a^d(T)$ (kJ/mol) of the various polar solvents adsorbed on PLA polymer as a function of the temperature.

T(K)	CH_2Cl_2	Ethyl Acetate	Acetone	Toluene	THF
313.15	3.567	4.219	2.880	5.032	3.640
318.15	3.513	4.155	2.836	4.956	3.585
323.15	3.465	4.098	2.797	4.888	3.536
328.15	3.363	3.977	2.714	4.744	3.431
333.15	3.261	3.856	2.632	4.600	3.327
338.15	3.237	3.828	2.612	4.566	3.302
343.15	3.219	3.806	2.598	4.540	3.284
348.15	3.447	4.077	2.782	4.863	3.517
353.15	3.682	4.354	2.972	5.194	3.757
358.15	3.904	4.617	3.151	5.508	3.984

363.15	4.133	4.888	3.336	5.830	4.217
368.15	3.928	4.646	3.171	5.542	4.008
373.15	3.724	4.404	3.006	5.253	3.800

Table S5. Values of -$\Delta G_a^0(T)$ (kJ/mol) of the various polar solvents adsorbed on PLA polymer as a function of the temperature.

T(K)	CH_2Cl_2	Ethyl Acetate	Acetone	Toluene	THF
313.15	13.209	7.574	7.976	14.753	12.424
318.15	12.493	6.930	7.711	14.325	11.910
323.15	11.782	6.312	7.431	13.905	11.403
328.15	10.999	5.660	7.035	13.455	10.840
333.15	10.215	5.077	6.862	13.005	10.277
338.15	11.223	7.055	9.825	13.911	12.447
343.15	12.237	8.850	13.578	14.825	14.640
348.15	11.196	8.242	10.325	14.300	12.273
353.15	10.160	7.021	6.647	13.784	9.499
358.15	10.268	6.828	6.205	13.957	9.417
363.15	10.383	6.713	5.990	14.138	9.341
368.15	10.032	6.229	5.475	13.664	8.820
373.15	9.658	5.669	5.011	13.240	8.235

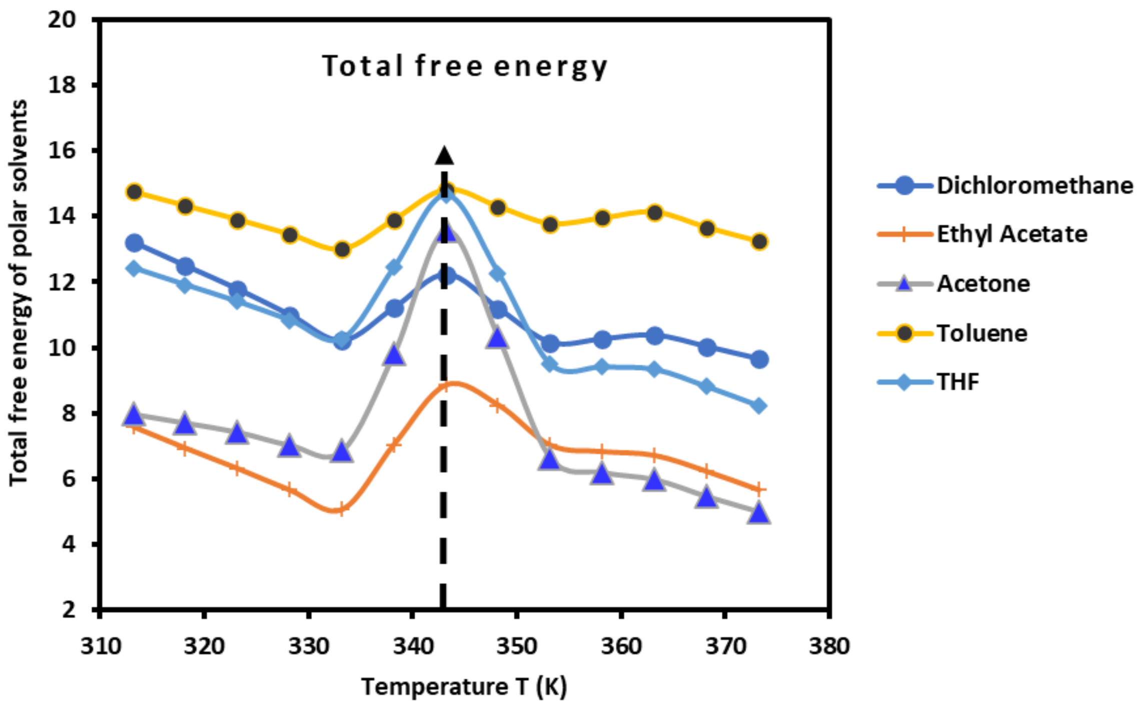

Figure S1. Variations the total free energy $\Delta G_a^d(T)$ (kJ/mol) of polar molecules adsorbed on PLA polymer as a function of the temperature.

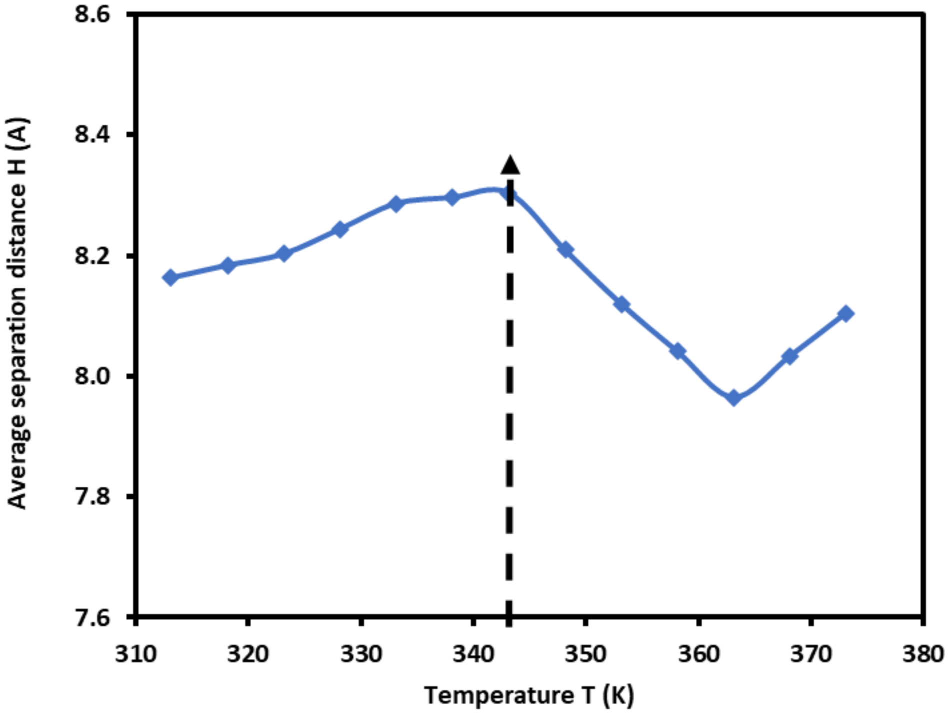

Figure S2. Variations of the average separation distance H (in Å) as a function of the temperature

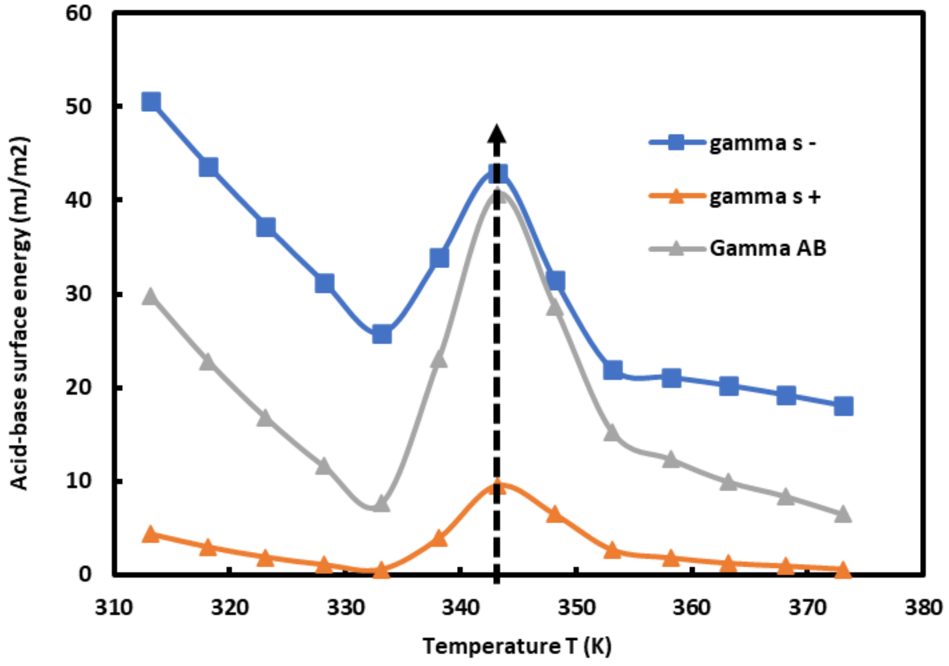

Figure S3. Variations of the polar acid and base surface energies γ_s^+, γ_s^- and γ_s^{AB} (mJ/m^2) of PLA as a function of the temperature.
